# Increasing levels of the endocannabinoid 2-AG is neuroprotective in the 1-methyl-4-phenyl-1,2,3,6-tetrahydropyridine mouse model of Parkinson's disease

**DOI:** 10.1016/j.expneurol.2015.07.024

**Published:** 2015-11

**Authors:** Ross B. Mounsey, Sarah Mustafa, Lianne Robinson, Ruth A. Ross, Gernot Riedel, Roger G. Pertwee, Peter Teismann

**Affiliations:** a Institute of Medical Sciences, University of Aberdeen, Foresterhill, Aberdeen, AB25 2ZD Scotland, UK; b Ninewells Hospital and Medical School, University of Dundee, Dundee, DD1 9SY Scotland, UK; c Department of Pharmacology and Toxicology, University of Toronto, Medical Sciences Building, 1 King's College Circle, Toronto, Ontario M5S 1A, Canada

**Keywords:** 2-AG, 2-arachidonoylglycerol, BOS, base of support, CB, cannabinoid, CE, coefficient of error, CV, coefficient of variance, COX-2, cyclo-oxygenase-2, DOPAC, 3,4-dihydrophenylacetic acid, ECS, endocannabinoid system, FAAH, fatty acid amide hydrolase, HPLC, high-performance liquid chromatography, IL, interleukin, MAGL, monoacylglycerol lipase, MPP^+^, 1-methyl-4-phenylpyridinium, MPTP, 1-methyl-4-phenyl-1,2,3,6-tetrahydropyridine, PD, Parkinson's disease, PPAR, peroxisome proliferator-activated receptor, SNpc, substantia nigra pars compacta, TH, tyrosine hydroxylase, TNF-α, tumor necrosis factor-alpha, Endocannabinoids, Parkinson's disease, MPTP, Neuroprotection

## Abstract

Parkinson's disease (PD) is a common chronic neurodegenerative disorder, usually of idiopathic origin. Symptoms including tremor, bradykinesia, rigidity and postural instability are caused by the progressive loss of dopaminergic neurons in the nigrostriatal region of the brain. Symptomatic therapies are available but no treatment slows or prevents the loss of neurons. Neuroinflammation has been implicated in its pathogenesis. To this end, the present study utilises the 1-methyl-4-phenyl-1,2,3,6-tetrahydropyridine (MPTP) neurotoxin to reproduce the pattern of cell death evident in PD patients. Herein, the role of a potential regulator of an immune response, the endocannabinoid system (ECS), is investigated. The most prevalent endocannabinoid, 2-arachidonoylglycerol (2-AG) (3 and 5 mg/kg), was added exogenously and its enzymatic degradation inhibited to provide protection against MPTP-induced cell death. Furthermore, the addition of DFU (25 mg/kg), a selective inhibitor of inflammatory mediator cyclooxygenase-2 (COX-2), potentiated these effects. Levels of 2-AG were shown to be upregulated in a time- and region-specific manner following MPTP administration, indicating that the ECS represents a natural defence mechanism against inflammation, potentiation of which could provide therapeutic benefits. The results expand the current understanding of the role that this signalling system has and its potential influence in PD.

## Highlights

• We investigate the role of 2-AG in a model of Parkinson's disease.
• 2-AG and inhibition of its metabolism by JZL184 protected against MPTP toxicity.
• A combination of JZL184 with a COX-2 inhibitor increased the effect.
• Manipulation of endocannabinoid levels might be useful for Parkinson's disease.

## 1 Introduction

Parkinson's disease (PD) is the second most common neurodegenerative disease in the world after Alzheimer's disease, with 0.3% of the population affected in industrialised countries; its prevalence increasing with age (de Lau and Breteler, 2006). It is characterised by motor abnormalities including tremor, muscle rigidity, paucity of voluntary movements and postural instability. The main neuropathological feature is the loss of dopaminergic neurons in the substantia nigra pars compacta (SNpc) and their projections to the striata (Dauer and Przedborski, 2004), although the exact cause of this degeneration is not certain in the majority of cases. Certain processes have been implicated in the generation of the disease, however. Substantial evidence supports the view that inflammation plays a pivotal role in the death of neurons in the basal ganglia. Activated forms of the resident immune cells of the central nervous system, microglia, have been found in the brains of PD patients at post-mortem (McGeer et al., 1988), while elevated levels of pro-inflammatory cytokines such as tumor necrosis factor-alpha (TNF-α), interleukin (IL)-1β and IL6 have been measured in the cerebrospinal fluid of patients (Mogi et al., 1994a, Mogi et al., 1994b, Blum-Degen et al., 1995, Muller et al., 1998).

The endocannabinoid system (ECS) is a signalling pathway composed of at least two G-protein coupled receptors (cannabinoid (CB)_1_ and CB_2_) and their endogenous ligands, involved in functions ranging from movement control to analgesia and emotional-affecting roles. The ECS has been targeted in models of various neurodegenerative diseases due to its potential in immune regulation: Alzheimer's disease (Ramirez et al., 2005, van der Stelt et al., 2006), Huntington's disease (Lastres-Becker et al., 2003, Sagredo et al., 2007, Dowie et al., 2010, Blazquez et al., 2011) and amyotrophic lateral sclerosis (Bilsland et al., 2006) have all been targeted with neuroprotective results. Exogenous addition of the most ubiquitous of the natural cannabinoid ligands, 2-arachidonoylglycerol (2-AG), has been shown to limit expression of pro-inflammatory mediator cyclooxygenase-2 (COX-2) (Zhang and Chen, 2008), possibly through activation of CB_1_ receptors, thereby protecting neurons. 2-AG has previously proven to be neuroprotective (Panikashvili et al., 2005) and is concentrated in the areas affected by PD (Bisogno et al., 1999), making it an attractive therapeutic target.

The two major endocannabinoids have their signalling tightly controlled by enzymatic activity. Each has a distinct pathway with different enzymes involved: *N*-arachidonoylethanolamine (anandamide) is primarily metabolised by fatty acid amide hydrolase (FAAH), while 2-AG is principally degraded by monoacylglycerol lipase (MAGL) (Dinh et al., 2002). A study of serine hydrolases in the mouse brain ascribed around 85% of 2-AG metabolism to this enzyme (Blankman et al., 2007). This study utilises the *N*-aryl carbamate URB602 and the more recently synthesised JZL184, as these have been found to be both potent and selective inhibitors of MAGL (King et al., 2007, Long et al., 2009b, Long et al., 2010, Pan et al., 2009) and therefore suitable for use in manipulation of the ECS. In addition, a selective COX-2 inhibitor, DFU, was used to determine whether the effects of exogenous 2-AG administration could be amended or potentiated, while also providing mechanistic information about the endocannabinoid. Herein we investigated if increasing levels of 2-AG *via* its exogenous addition or inhibition of its metabolic enzyme would lead to neuroprotection in the 1-methyl-4-phenyl-1,2,3,6-tetrahydropyridine hydrochloride (MPTP) mouse model.

## 2 Methods

### 2.1 Animals and treatment

Twelve week-old male C57BL6/J mice (Charles River Laboratories, UK; 4–12 per time point) received intraperitoneal (i.p.) injections of MPTP (SigmaAldrich, Poole, UK) at 30 mg/kg freebase dissolved in 0.9% saline solution — one injection per day for five consecutive days, before being sacrificed by decapitation at 21 days after the last injection. Control mice received saline only. This treatment was in accordance with published guidelines (Jackson-Lewis and Przedborski, 2007). All procedures were in accordance with the Animals Scientific Procedures Act (1986) and approved by the Home Office (Dundee, UK). Mice were housed in appropriately sized cages with access to food and water *ad libitum*, with a 12-hour light–dark cycle (lights on at 7 am). Experiments were carried out in accordance with the European Communities Council Directive (63/2010/EU) with local ethical approval, a project license under the UK Scientific Procedures Act (1986),

### 2.2 Drug treatments

2-AG (3 or 5 mg/kg), CP55,940 (0.5 mg/kg) (both Tocris Bioscience, Ellisville, MO), URB602 (10 mg/kg), JZL184 (40 mg/kg) (both Cayman Chemicals, Ann Arbor, MI) and DFU (25 mg/kg) (Merck, Damstadt, Germany) were dissolved *via* gentle sonification in a vehicle consisting of ethanol, chremophor EL (SigmaAldrich) and saline solution at a ratio of 1:1:18 v/v/v. All drugs were administered by i.p. injection 1 ×/day for 3 days prior to MPTP treatment, throughout the treatment and until sacrifice. Control mice received vehicle only. The amount of compound was chosen based on previous studies (2-AG: Panikashvili et al., 2001; CP55,940: Fontanellas et al., 2005; URB602: Comelli et al., 2007; JZL184: Nomura et al., 2011; and DFU: Riendeau et al., 1997).

### 2.3 Immunostaining and stereological counting

Brains were extracted and post-fixed in 4% paraformaldehyde (PFA) for 24 h before being cryoprotected in 30% sucrose in 0.1 M phosphate buffer. Brains were then snap frozen in 2-methylbutane and stored at − 80 °C until use. Sections of the SNpc and striatum were cut at a thickness of 30 μm on a Leica CM1900 cryostat (Leica Microsystems GmbH Wetzlar, Germany) and placed in 24- or 48-well plates in 0.1 M PBS containing 0.1% sodium azide. Immunostaining was carried out using the free-floating technique, as described previously (Sathe et al., 2012), with primary antibody incubation taking place at 4 °C for 48 h with rabbit anti-tyrosine hydroxylase (TH; 1:1000; Millipore, Watford, UK) in 2% normal goat serum. Sections were washed in 0.1 M PBS, mounted on gelatinised slides and counter-stained with Nissl reagent and cover-slipped with Entellan (Merck). Counting of TH- and Nissl-positive cells was performed using regular light microscopy (AxioImager M1, Carl Zeiss, Hertfordshire, UK) and the optical fractionator method (West, 1993) (Stereo Investigator Version 7, BMF Bioscience, Magdeburg, Germany).

### 2.4 High performance liquid chromatography analysis

High-performance liquid chromatography (HPLC) with electrochemical detection was used to measure striatal levels of dopamine and 3,4-dihydroxyphenylacetic acid (DOPAC) (Sathe et al., 2012).

### 2.5 Striatal 1-methyl-4-phenylpyridinium iodide (MPP^+^) levels

Liquid chromatography with on-line ultraviolet detection/tandem mass spectrometry (LC–UV–MS–MS) was used to measure striatal levels of MPP^+^ following drug treatments. Briefly, mice received treatment as described above and, 90 min after a single MPTP injection (30 mg/kg), mice were sacrificed. The striata were dissected out and snap frozen on solid carbon dioxide. Striata were then prepared as described above. Following centrifugation, 2 μl of sample was injected onto a Hichrom 5 μm C18 column (Hichrom, Theale, UK). The mobile phase consisted of 80% 0.1% formic acid in water/20% 0.1% formic acid in acetonitrile. Flow rate was 0.2 ml/min. MPP^+^ was detected by a photodiode array detector set to 295 nm, and a triple quadrupole mass spectrometre with a mass to charge ratio of 170–128 at 32 V and 1.9 mTorr (ThermoSurveyor PDA/TSQ Quantum, ThermoScientific, Loughborough, UK). Data were collected and processed using Xcalibur 2.0.7 SP1.

### 2.6 Home cage observation

Locomotor activity was recorded using 32 Activity Cages (Ugo Basile, Varese, Italy) placed in a quiet holding room. They consist of two rows of horizontal infra-red beams (1 cm distance) and monitor horizontal ambulations and vertical rearings as number of beam breaks. Animals were placed into freshly saw-dusted Perspex home cages (42 cm × 26.5 cm × 15 cm). Animals were placed into the novel cage and allowed to freely explore the environment for 60 min. Beam breaks due to horizontal and vertical movements were automatically recorded and transmitted by an interface and online displayed using Activity software (Ugo Basile). Locomotor activity was monitored continuously in 1 minute intervals and then pooled for a period of 60 min. In addition the number of faecal balls was noted for the observation period as a measure of anxiety.

### 2.7 Gait analysis (CatWalk)

Gait analysis was conducted using the CatWalk system (CatWalk 7.1, Noldus IT, Wageningen, Netherlands) in a dimly lit room. The apparatus consists of an enclosed walkway (50 cm in length) consisting of LED-illuminated glass and an under-floor camera for recording footfalls and footprint patterns. Recordings were digitised and autoanalysed using CatWalk 7.1 software (Noldus IT). The mice were placed at one end of the walkway and left to cross to the other end freely. They were removed only after they had made not less than three compliant runs, *i.e.*, subjects crossed the walkway in less than 5 s. Two infrared beams were used to detect the arrival of the mouse at each end and controlled (start/stop) data acquisition. Endpoints included relative paw position (hind overlapping/non-overlapping with front paws), timing (stand, swing and step cycle), base of support, pressure and dimensions of each footfall, which enabled calculation of stride length, paw distance, and regularity index (preferred paw placement sequence during walking) and were averaged for all compliant runs. The apparatus was cleaned with 70% ethanol between animals.

### 2.8 Statistical analysis

Data were analysed in GraphPad Prism 5 for Windows (GraphPad Software, La Jolla, CA). All values are expressed as the mean ± SEM. Normal distribution of the data was tested and the homogeneity of variance confirmed with the Kolmogorov–Smirnov test. For data sets, one-way analysis of variance (ANOVA) was used to analyse differences among means with time, treatment or genotype as the independent factor, when the data was normally distributed. When ANOVA showed significant differences *post hoc* testing was used to make comparisons between means: Dunnett's *post hoc* test was used for time-course studies and Student–Newman–Keuls *post hoc* test was used to make pair-wise comparisons in all other studies. The null hypothesis was rejected at the 0.05 level. Outliers were eliminated based on descriptive statistics performed by SPSS (IBM SPSS Statistics Version 21 for Windows, Hampshire, UK). To assess sampling strategy and homogeneity of the group coefficient of variance (CV) and coefficient of error (CE) (Gundersen and Jensen, 1987) were assessed.

## 3 Results

### 3.1 2-AG attenuates MPTP-induced degeneration of nigrostriatal neurons but not in a dose-dependent manner

**Fig. 1:**
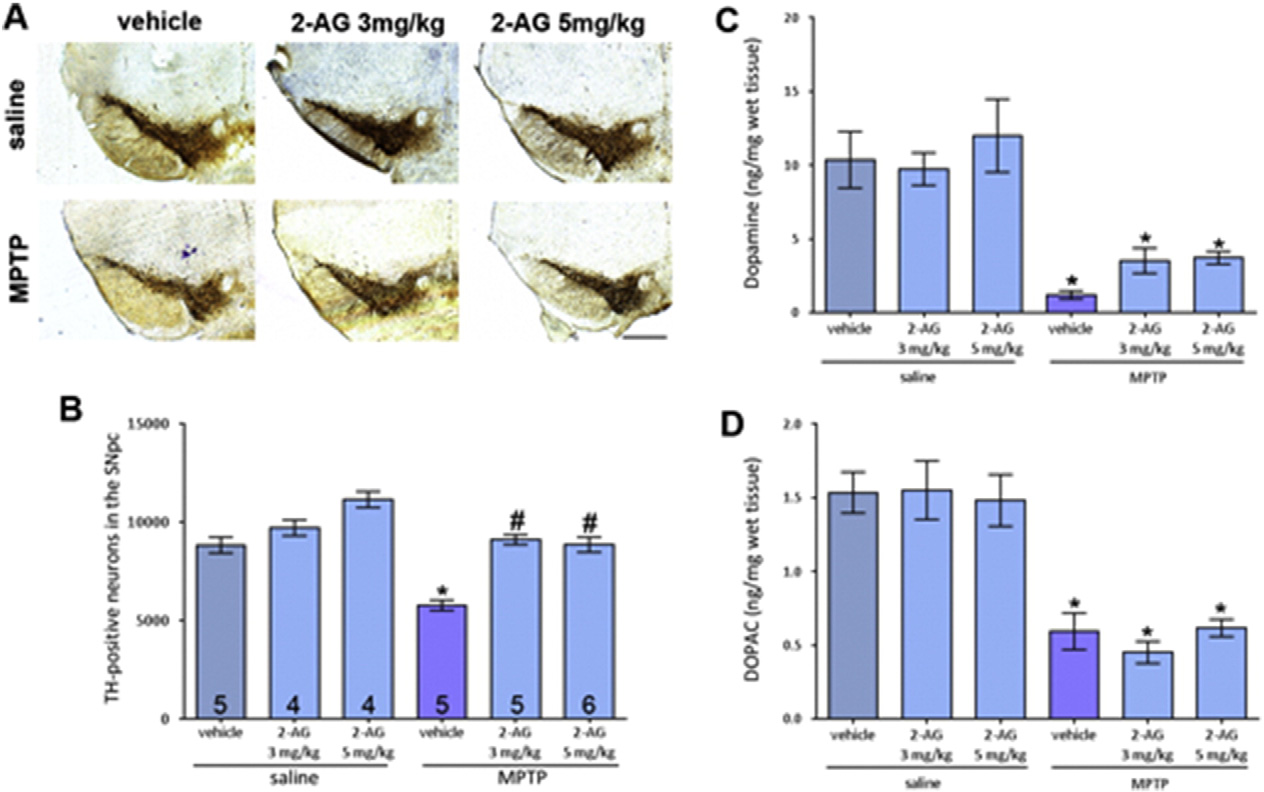
Effect of two doses of 2-AG on nigral MPTP neurotoxicity. (A) Representative photomicrographs of TH-stained SN sections following treatment with 3 mg/kg or 5 mg/kg 2-AG with either saline or MPTP (scale bar is 200 μm). (B) Both doses of 2-AG similarly attenuated MPTP-induced loss of TH-positive neurons but the protective effect did not translate to protection of striatal dopaminergic fibres (C) or striatal dopamine content (D). Data are mean ± SEM, n — numbers displayed on bars. One-way ANOVA: *F*(5, 23) = 3.998, *p* = 0.0093. * *p* < 0.05, compared to equivalent saline-treated group; # *p* < 0.05, compared to vehicle and MPTP group (Newman–Keuls *post hoc* test).

TH, the enzyme responsible for the rate-limiting step in the synthesis of dopamine, was stained. It was found that the specified doses of 2-AG provided equal levels of neuroprotection when assessing TH-positive neuron numbers in the SNpc through stereological counting (Fig. 1A and B). 2-AG alone did not affect neuronal survival. The number of TH-positive cells was reduced by 35.8% following sub-acute MPTP administration compared to saline-treated control brains. This reduction was attenuated by both 3 mg/kg and 5 mg/kg 2-AG. There were some changes found between groups when Nissl, a non-specific neuronal marker, was used to stain cells, with 3 mg/kg 2-AG and saline, and MPTP with vehicle, or 3 mg/kg 2-AG showing significant deviations from control numbers (Fig. 1B). Statistics on CV and CE can be found in Supplemental Table 1.

The effect of 2-AG on striatal innervation by dopaminergic fibres was evaluated by measuring TH immunoreactivity, giving an indication of the density of striatal fibres. Both doses of 2-AG failed to significantly attenuate the MPTP-induced reduction in TH immunoreactivity although an increase in mean density is evident.

Treatment with the CB_1_/CB_2_ receptor agonist CP55,940 and the endocannabinoid 2-AG (5 mg/kg) did not produce attenuation of striatal dopamine loss induced by MPTP at levels considered to be significant (Fig. 1C; and MPTP + vehicle: 0.98 ± 0.33 ng/mg wet tissue weight; MPTP + CP55,940: 2.06 ± 0.31 ng/mg wet tissue weight). The same was the case for DOPAC (Fig. 1D; MPTP + vehicle: 0.59 ± 0.12 ng/mg wet tissue weight; MPTP + CP55,940: 0.62 ± 0.09 ng/mg wet tissue weight).

Since mice were receiving drugs as pre- and post-treatments, it was important to ensure that these did not interfere with the metabolism of MPTP into the active toxin MPP^+^. Liquid chromatography with ultra-violet detection and tandem mass spectrometric detection was used to measure the striatal content of the toxin following infusion of the drug and MPTP to check that drugs did not interfere with the bio-activation of the toxin (Table 1). Data show no change in striatal MPP^+^ levels across the treatments.

### 3.2 Significant neuroprotection following inhibition of MAGL by JZL184 after MPTP-induced degeneration of nigrostriatal neurons but not URB602

**Fig. 2:**
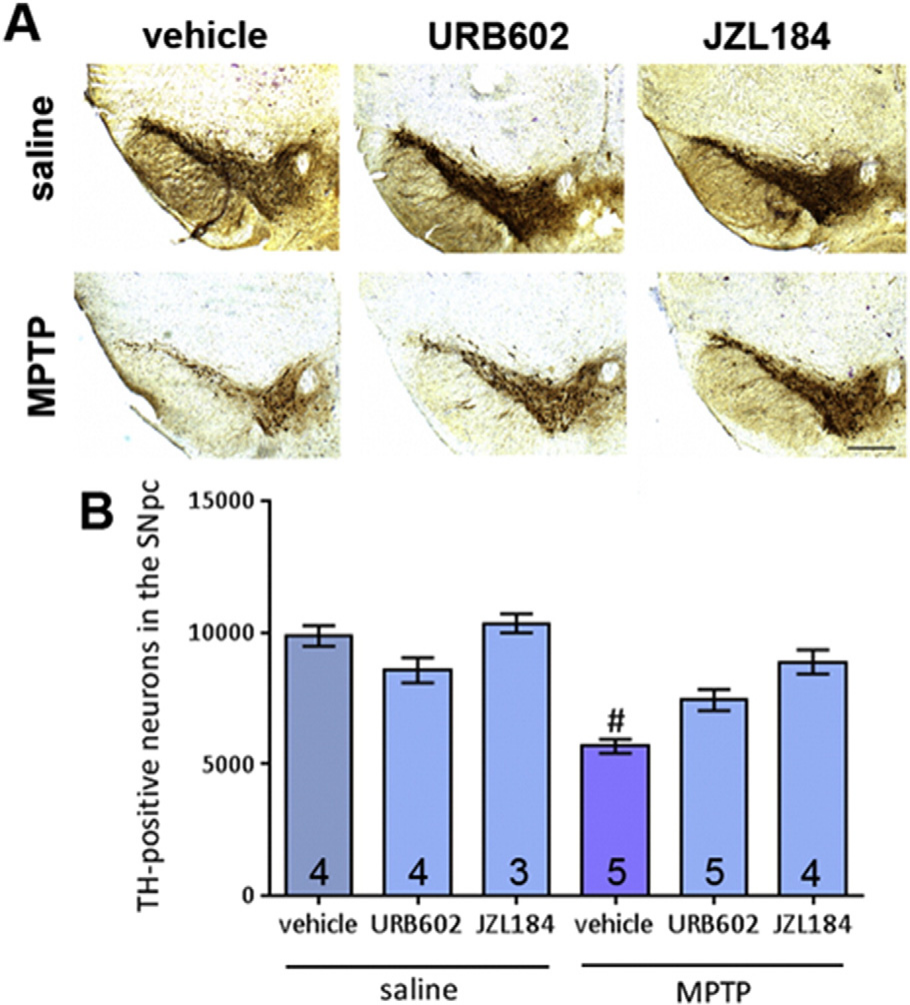
Effect of two MAGL inhibitors, URB602 and JZL184, on MPTP toxicity in the SNpc. (A) Representative photomicrographs of TH-stained SN sections following treatment with URB602 or JZL184 with either saline or MPTP (scale bar is 200 μm). (B) Neither treatment significantly attenuated MPTP-induced reductions in TH-positive neurons. Data are mean ± SEM, n — numbers displayed on bars. One-way ANOVA: *F*(5, 19) = 3.500, *p* = 0.02. # *p* < 0.05, compared to saline treated control groups and JZL184 + MPTP group (Newman–Keuls *post hoc* test).

MAGL inhibitors were utilised to increase 2-AG levels through inhibition of its selective metabolic enzyme, and thereby provide another means of increasing levels of the endocannabinoid besides exogenous addition. URB602 administration did not lead to an attenuation of MPTP-induced reduction in TH-positive cell numbers. On the other hand JZL184 administration led to significantly higher TH-positive cell numbers after MPTP (Fig. 2A and B). Statistics on CV and CE can be found in Supplemental Table 2.

### 3.3 DFU augments the neuroprotection provided by JZL184

Since exogenous 2-AG has previously been found to mediate protection from excitotoxicity in hippocampal neurons through inhibition of COX-2 expression (Zhang and Chen, 2008), it was considered that this may be the mechanism of action by which dopaminergic neurons are protected in the MPTP model. To test this theory, the selective COX-2 inhibitor DFU (25 mg/kg) was administered to mice by i.p. injection. In addition, JZL184 (40 mg/kg) was added in combination with DFU to see if the neuroprotective potential it shows alone can be potentiated by the co-administration of this agent.

**Fig. 3:**
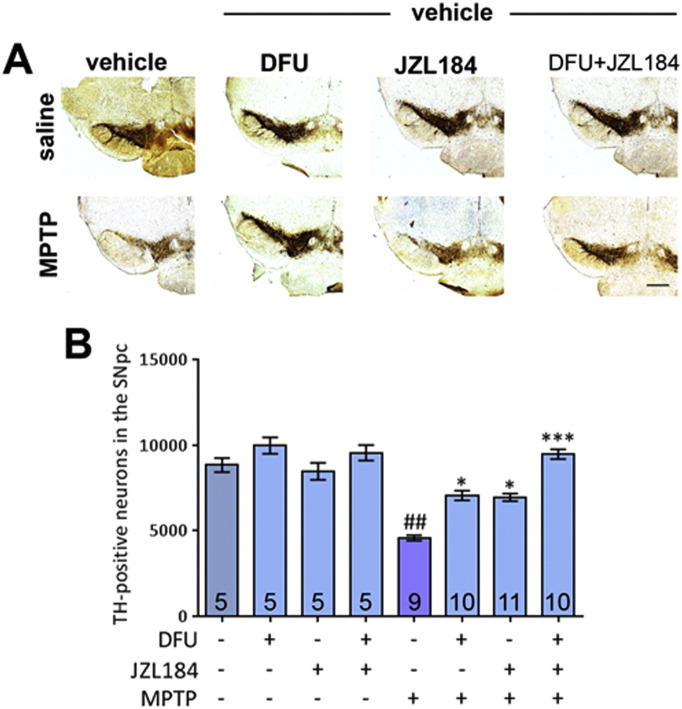
Effect of dual MAGL and COX-2 inhibition on MPTP neurotoxicity. (A) Representative photomicrographs of TH-stained SN sections following treatment with DFU and/or JZL184 with either saline or MPTP (scale bar is 200 μm). (B) MPTP causes a reduction in TH-positive nigral neurons. This is attenuated by treatment with DFU or JZL184 alone and, when administered as a co-treatment, the drugs result in a pronounced effect, providing recovery to saline TH levels. Data are mean ± SEM, n — numbers displayed on bars. Experiment was performed twice and the results pooled. One-way ANOVA: *F*(7, 52) = 5.129, *p* = 0.0002. ## *p* < 0.01 compared to vehicle and saline group; * *p* < 0.05, *** *p* < 0.001, compared to vehicle and MPTP group (Newman–Keuls *post hoc* test).

As expected, MPTP caused a highly significant death-rate among TH-positive neurons in the SNpc, with around 50% of these neurons lost (Fig. 3A and B). This is attenuated by treatment with DFU or JZL184 alone. However, treatment with the two agents in combination produced additive benefits, leading to total recovery of TH-positive neuron numbers to saline levels. Statistics on CV and CE can be found in Supplemental Table 3.

**Fig. 4:**
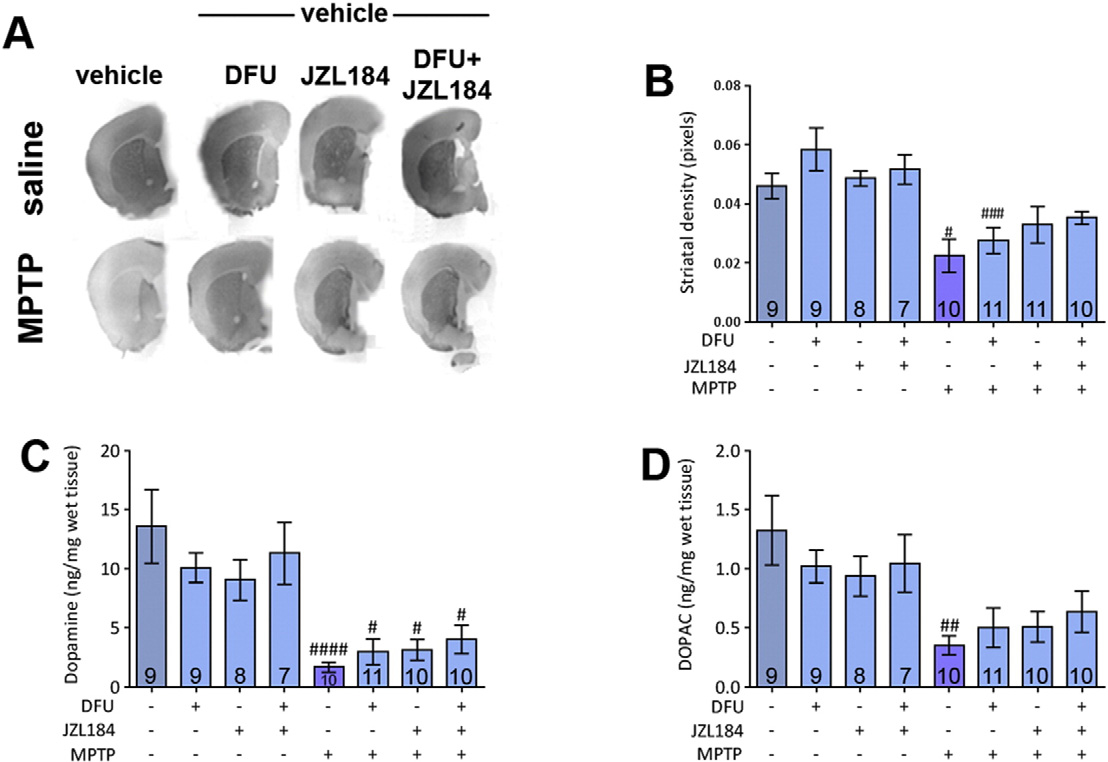
Striatal density and HPLC analysis of dopamine and DOPAC levels following MPTP treatment and administration of DFU and JZL184. (A) Representative scanned images of striatal sections following drug treatments. (B) No attenuation of MPTP-induced decreases in striatal density is shown. DFU and co-treatment samples are significantly lower than their saline-treated equivalents. Experiment was performed twice and the results pooled. One-way ANOVA: *F*(7, 67) = 6.339, *p* = 0.0152. (C) Dopamine levels of all treatments are significantly lower than their saline-treated equivalents, but addition of DFU or JZL184 does not significantly attenuate this reduction. One-way ANOVA: *F*(7, 67) = 7.748, *p* < 0.0001. (D) DOPAC levels are reduced after MPTP treatment. Other values are not significantly altered. One-way ANOVA: *F*(7, 67) = 3.603, *p* = 0.0024. Data are mean ± SEM, n — numbers displayed on bars. Experiments were performed twice and the results pooled. # *p* < 0.05, ## *p* < 0.01, #### *p* < 0.0001, compared to an equivalent saline-treated group (Newman–Keuls *post hoc* test).

Striatal dopaminergic innervation was again evaluated by measuring the optical density of TH immunoreactivity. There was no significant attenuation of MPTP-induced decreases in striatal density when DFU or JZL184 was administered, although there is a trend towards this result following a similar pattern to nigral TH protection. But only immunoreactivity values of MPTP only and MPTP + DFU treated brains were significantly lower than their equivalent saline-treated samples (Fig. 4A and B).

HPLC analysis was used to determine the levels of dopamine and DOPAC in the striatum following treatments. MPTP induced a significant reduction in striatal dopamine levels (Fig. 4C), but none of the treatments succeeded in attenuating this to a significant level. This situation is mirrored when striatal levels of DOPAC were measured (Fig. 4D)

### 3.4 The behaviour of the mice is not affected when assessed 26 days after MPTP

**Fig. 5:**
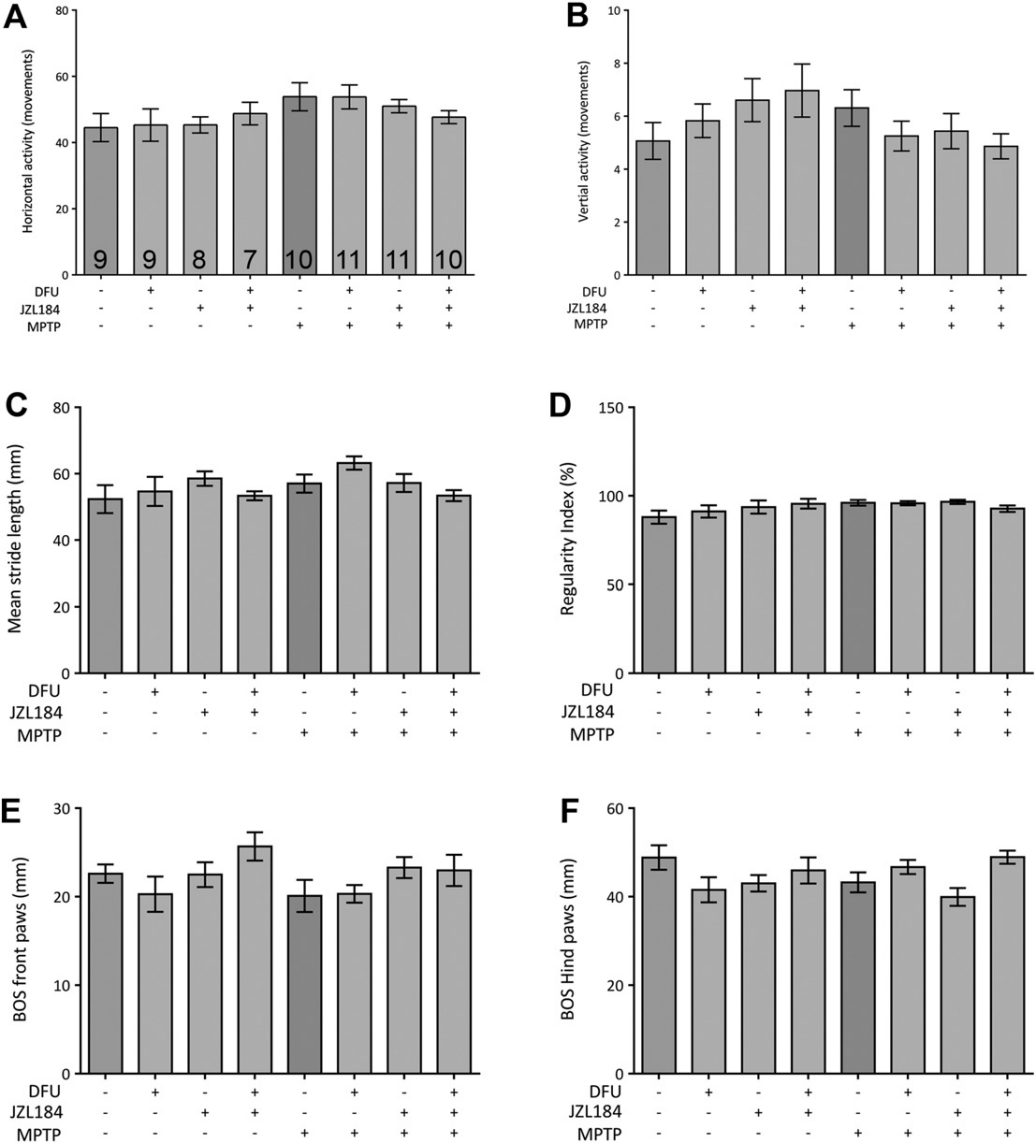
Activity and gait-related parameters were not affected by MPTP. Global activity during 60 min home cage exploration yielded no difference in horizontal (A) or vertical movements (B). Treatment with MPTP, DFU or JZL184 does not affect the mean stride length (C) and the proportion of normal step patterns (D). Similarly, there was no effect on base of support (BOS) from either the front (E) or hind (F) paws. Data are mean ± SEM, n = 7–11 per group.

Behavioural analysis was undertaken to assess whether treatments would induce behavioural abnormalities in the mice. Tests were performed 26 days after MPTP exposure to establish full-blown PD in mice. Neither activity-related parameters (vertical and horizontal movement) nor gait-related proxies of the Catwalk (stride length, regularity index or base of support) differed between cohorts (Fig. 5). Although there was some variation between groups, the most consistent feature was a small reduction in gait (base of support) for both front and hind paws in the MPTP cohort (Fig. 5E,F). However, despite severe frank cell loss in the substantia nigra, animals were not motorically impaired when exposed to MPTP.

### 3.5 2-AG is released in a region- and time-specific manner

To find whether the endocannabinoid system represents a natural defence mechanism against the specific deleterious effects that the MPTP model induces in mice, levels of endogenous 2-AG were measured by LC–MS–MS at several time-points following a chronic treatment regimen which mirrors that administered to the mice in the previous treatment studies.

**Fig. 6:**
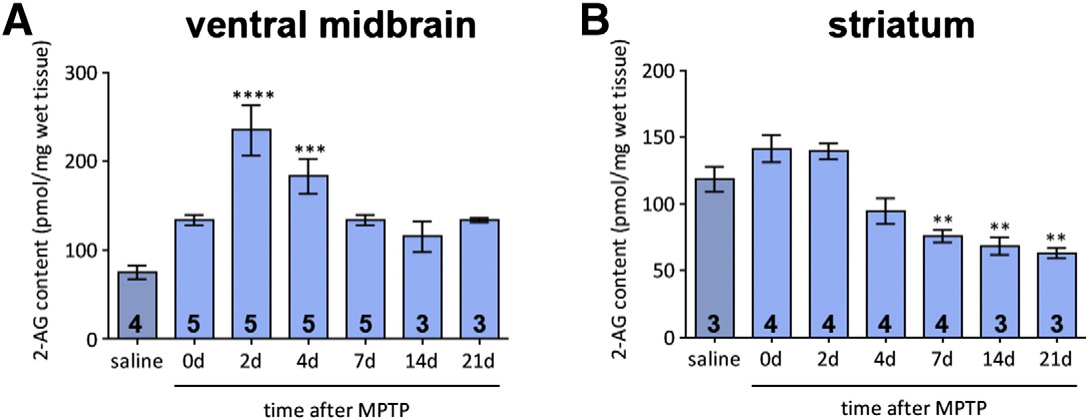
Time-course of 2-AG levels measured by mass spectrometry in the ventral midbrain and striatum of mice following sub-chronic MPTP treatment. (A) 2-AG levels in the ventral midbrain increase following MPTP administration, showing a significant increase between 2 and 4 days after treatment, before returning to control levels. One-way ANOVA: *F*(6, 23) = 9.881, *p* < 0.0001. (B) Striatal levels of 2-AG show no initial increase and decline steeply between 7 and 21 days after MPTP treatment. One-way ANOVA: *F*(6, 22) = 16.80, *p* < 0.0001. Data are mean ± SEM, n — numbers per time-point displayed on bars. ** *p* < 0.01, *** *p* < 0.001, **** *p* < 0.0001, compared to saline group (Dunnett's *post hoc* test) (d — days following the end of MPTP treatment (30 mg/kg daily administration)).

Following MPTP administration, levels of 2-AG in the ventral midbrain (Fig. 6A) are increased significantly by 2 days after the end of MPTP administration. A significant upregulation of the endocannabinoid is also evident 4 days after MPTP but levels return close to control after 7 days. The situation in the striatum is different (Fig. 6B). There is initially no change in 2-AG levels following MPTP administration followed by a decline which reaches levels significantly lower than those seen in control tissues at 7 days after treatment. These low levels are maintained until the end of the study at 21 days.

## 4 Discussion

Our data show that increasing levels of the endocannabinoid 2-AG, either by its exogenous addition or inhibiting its metabolism by using selective inhibitors of this enzymatic activity, is protective against the neurodegenerative effects induced by the neurotoxin MPTP. This effect can be augmented by the addition of the selective COX-2 inhibitor DFU.

Treatment with 2-AG in mice attenuated the loss of TH-positive neurons but not striatal levels of dopamine caused by the chronic regimen of MPTP. The level of protection may be higher with an increased dose. Although the dose-dependent treatment shown here showed no significant changes in protection against MPTP toxicity between 3 mg/kg and 5 mg/kg, a higher dose may produce protection, as suggested by the effect of higher 2-AG levels resulting from chronic treatment with the selective MAGL inhibitor JZL184. Higher levels of 2-AG, due to MAGL inhibition by JZL184 have also been implicated in synaptic and cognitive improvements in a model of Alzheimer's disease (Zhang et al., 2014).

The CB_1_/CB_2_ receptor agonist CP55,940 caused a small, but not significant increase in the levels of striatal dopamine following MPTP insult. It seems likely that activation of CB_1_ is required for neuroprotection (Chiarlone et al., 2014, Blazquez et al., 2015). The issue of whether activation of CB_1_ is protective or harmful to neurons is one which has provided much debate, with a number of different perspectives supporting the notion that CB_1_ receptor activation is neuroprotective (Melis et al., 2006, Iuvone et al., 2007, Chen et al., 2011), although other studies argue that neuroprotection is CB_1_ receptor activation-independent or dependent on its blockade (Nomura et al., 2011, Lastres-Becker et al., 2005, Kelsey et al., 2009, Mukhopadhyay et al., 2010, Garcia et al., 2011). Another study shows that activation of CB_2_ receptors is crucial to the neuroprotection provided by the non-selective agonist WIN55,212-2 (Price et al., 2009). Expression of CB_2_ receptors, previously thought to only exist in peripheral areas (reviewed by Atwood and Mackie, 2010), is upregulated at times of stress and immune reaction, particularly in microglia. Activation of CB_2_ receptors has been shown in models of Huntington's disease to be effective against many of the same pathological changes which occur in PD, including microglial activation and neuroinflammation (Palazuelos et al., 2009). These pathways may be initiated by specific activation of the two previously cloned receptors as well as those pharmacologically characterised, but not yet cloned (reviewed by Mackie and Stella, 2006). An example of this activation of these “new players” is that of the CB_2_ receptor and an abnormal-cannabidiol-sensitive receptor, which has been shown to provide neuroprotection by preventing the accumulation of microglia (Kreutz et al., 2009). It has previously been suggested that 2-AG protects neurons by inhibiting the expression of the inflammatory enzyme COX-2 (Zhang and Chen, 2008). 2-AG is also a substrate for COX-2 (Kozak et al., 2000), which metabolizes 2-AG into potentially harmful prostaglandins: a process that may exacerbate any inflammation and degeneration. 2-AG can also directly reduce the levels of reactive oxygen species, cytokines and prostaglandin E_2_ (Zhang and Chen, 2008). Therefore, limiting the expression of COX-2 would on the one hand prevent the accumulation of possible inflammatory mediators generated by COX-2-mediated 2-AG metabolism and on the other hand more 2-AG would be available for possible neuroprotective effects mediated *via* cannabinoid receptors. Zhang and Chen (2008) also showed that the inhibition of COX-2 expression was dependent on the CB_1_ receptor. This evidence, along with that outlined above, indicates that the most successful utilisation of the ECS in neuroprotection could result from non-selective activation of the cannabinoid receptors, making 2-AG a useful therapeutic agent to meet this aim since 2-AG can confer neuroprotection through its anti-inflammatory and anti-oxidant properties. To take full advantage of all the numerous neuroprotective properties that 2-AG has shown, including anti-inflammatory and antioxidant, a manipulation of more than one of the cannabinoid receptors is required. 2-AG seems suitable as a treatment option, not only is it a potent and abundant agonist of both cannabinoid receptors as it is a natural ligand, and thus avoids the potential complications which can occur with exogenously administered synthetic agonists. In addition to this, the peroxisome proliferator-activated receptor (PPAR)-γ has been shown to be crucial in some of the neuroprotective effects produced by 2-AG (Du et al., 2011). Thus, concurrent use of a PPARγ receptor agonist, such as rosiglitazone, may potentiate the benefits of 2-AG in the MPTP model.

An alternative to the exogenous addition of 2-AG is the inhibition of its metabolic enzyme, MAGL. We utilised JZL184, a potent inhibitor of this enzyme (Long et al., 2009a, Long et al., 2009b) alongside URB602, a more established inhibitor (Hohmann et al., 2005). However, another group has cast doubt on the potency of URB602 as a MAGL inhibitor and its preference over FAAH *in vitro* (Vandevoorde et al., 2007). URB602 has been shown to be less effective against MPTP toxicity in the present study, but whether this is as a direct result of the drug being an inferior inhibitor of MAGL, and therefore less effective at increasing 2-AG levels, is not clear. JZL184 in itself proved to be protective against MPTP-induced cell death. This is in line with other reports, which show the neuroprotective effect of JZL184 (Nomura et al., 2011, Fernandez-Suarez et al., 2014). In addition to inhibition of MAGL and thus increasing levels of 2-AG, it seems likely that JZL184 might itself act directly on the CB_2_ receptor, but not the CB_1_ receptor (Aymerich et al., 2015).

As pharmacological inhibition of MAGL has proven effective in attenuating MPTP-induced cell death, another way the effect of MAGL inhibition could be evaluated is the genetic ablation of MAGL by manipulation of *MgII*. However, this technique has been shown to reduce the expression and functional capacity of CB_1_ receptors (Schlosburg et al., 2010). This questions the long-term use of JZL184 as a viable treatment option, as the data from this study indicate that chronically elevated levels of 2-AG antagonise the brain ECS *via* CB_1_ adaptations (Schlosburg et al., 2010). Interestingly, a sustained pharmacological disruption of FAAH had no such effect. However, it is clear that activation of the ECS is important and effective in neuroprotection, therefore such full, functional incapacity should be avoided. Studies using other models have shown that the pharmacological elevation of endocannabinoid levels is effective against toxic insults, including β-amyloid toxicity when administering an inhibitor of cannabinoid re-uptake at sub-chronic levels (van der Stelt et al., 2006), so further work is merited in the MPTP model. An adapted treatment regime could be considered to avoid the potential problems outlined by Schlosburg et al. (2010) and discover whether JZL184 remains effective in a shorter treatment regimen and/or at a lower dose.

The combination of JZL184 with the specific COX-2 inhibitor DFU had a more pronounced effect on MPTP-induced toxicity. Previous studies have already shown that inhibition of COX-2 has a neuroprotective effect on MPTP-induced toxicity (Teismann et al., 2003). Interestingly, by using malonate it is reported that JZL184 leads to an increase of 2-AG levels, which in turn increases malonate-induced cell damage (Valdeolivas et al., 2013). A JZL184-induced increase in 2-AG was not shown to lead to neurotoxicity. But it might also be the case that in the models used the beneficial effects of JZL184 as well as 2-AG itself on the CB_2_ receptor outweigh the deleterious effects mediated by the 2-AG increase. The beneficial effects seen by the addition of the COX-2 inhibitor DFU, besides acting directly on COX-2 itself, might have been due to an attenuation of the pro-inflammatory prostaglandin E2-G.

We found that the protection on dopaminergic neurons in the SNpc due to DFU and/or JZL184 did not extend to the striatal fibres. But this is in accordance with other studies, where protection of dopaminergic somata against MPTP-induced cell death did not extend to the striatal dopaminergic fibres (Dehmer et al., 2000, Tieu et al., 2004, Liberatore et al., 1999). This might also explain why DFU and/or JZL184 treatment failed to attenuate any MPTP-induced behavioural changes. Although it is interesting to note that there was no significant difference between the MPTP group treated with JZL184 as well as the one treated with both DFU and JZL184 and their corresponding control group, putting it more in line with the findings of Fernandez-Suarez et al. (2014). Interestingly enough, it was shown that MPTP leads to an increase in CB_2_ expression in the SNpc but not the striatum (Price et al., 2009), thus the approach of affecting 2-AG levels might have a more substantial benefit in the nigra due to the increased presence of CB_2_.

In summary, our results show that the endocannabinoid 2-AG is neuroprotective in the MPTP mouse model. The benefits can be achieved both by exogenous addition of 2-AG and by specifically inhibiting the enzyme MAGL, which controls the ligand's metabolism, with the infusion of JZL184. Further studies using animals lacking CB_1_ or CB_2_ should verify if both CB_1_ and CB_2_ receptors are needed to ensure that the full spectrum of 2-AG neuroprotective properties can be achieved. Further work should focus on a more detailed delineation of the mechanism by which 2-AG works, and possible ways to potentiate its action.

The following are the supplementary data related to this article.

Supplemental Table 1 Coefficient of variation (CV) and coefficient of error of the mean (CE) for stereological assessment of TH-positive neurons in the substantia nigra.

Supplemental Table 2 Coefficient of variation (CV) and coefficient of error of the mean (CE) for stereological assessment of TH-positive neurons in the substantia nigra.

Supplemental Table 3 Coefficient of variation (CV) and coefficient of error of the mean (CE) for stereological assessment of TH-positive neurons in the substantia nigra.

## Figures and Tables

**Table 1:** Effect of drugs used on striatal MPP^+^ levels. No differences were seen in striatal levels of MPP^+^ between mice receiving drugs or those receiving saline. Data are mean ± SEM, n = 4–8 per group (one-way ANOVA followed by Newman–Keuls *post hoc* test; n.s.).

	Saline	2-AG	URB602	JZL184	DFU
MPP^+^ (μg/g wet tissue)	12.31 ± 2.90	9.62 ± 0.33	12.89 ± 0.38	13.39 ± 2.51	10.13 ± 0.88
