# Supplementary material for: Increasing levels of the endocannabinoid 2-AG is neuroprotective in the 1-methyl-4-phenyl-1,2,3,6-tetrahydropyridine mouse model of Parkinson's disease
Source: Exp Neurol. 2015 Nov;273:36–44. doi: 10.1016/j.expneurol.2015.07.024 (PMC4654430; doi:10.1016/j.expneurol.2015.07.024)
Supplement: Supplemental Table 1 — Coefficient of variation (CV) and coefficient of error of the mean (CE) for stereological assessment of TH-positive neurons in the substantia nigra. [file mmc1.docx]

Supplemental Table 1.

Coefficient of variation (CV) and coefficient of error of the mean (CE) for stereological assessment of TH-positive neurons in the substantia nigra

| Group | CV | CE |
| --- | --- | --- |
| vehicle + saline | 10.52 | 5.2 |
| 2-AG (3mg/kg) + saline | 10.28 | 5.25 |
| 2-AG (5mg/kg) + saline | 8.24 | 4.25 |
| vehicle + MPTP | 7.38 | 6.4 |
| 2-AG (3mg/kg + MPTP | 6.24 | 4.8 |
| 2-AG (5mg/kg + MPTP | 10.58 | 4.83 |
