# Supplementary material for: Increasing levels of the endocannabinoid 2-AG is neuroprotective in the 1-methyl-4-phenyl-1,2,3,6-tetrahydropyridine mouse model of Parkinson's disease
Source: Exp Neurol. 2015 Nov;273:36–44. doi: 10.1016/j.expneurol.2015.07.024 (PMC4654430; doi:10.1016/j.expneurol.2015.07.024)
Supplement: Supplemental Table 2 — Coefficient of variation (CV) and coefficient of error of the mean (CE) for stereological assessment of TH-positive neurons in the substantia nigra. [file mmc2.docx]

Supplemental Table 2.

Coefficient of variation (CV) and coefficient of error of the mean (CE) for stereological assessment of TH-positive neurons in the substantia nigra

| Group | CV | CE |
| --- | --- | --- |
| vehicle + saline | 10.50 | 5.33 |
| URB602 + saline | 8.88 | 5.25 |
| JZL184 + saline | 6.03 | 4.33 |
| vehicle + MPTP | 7.80 | 5.86 |
| URB602 + MPTP | 12.11 | 5.4 |
| JZL184 + MPTP | 10.41 | 4.8 |
