# Supplementary material for: Increasing levels of the endocannabinoid 2-AG is neuroprotective in the 1-methyl-4-phenyl-1,2,3,6-tetrahydropyridine mouse model of Parkinson's disease
Source: Exp Neurol. 2015 Nov;273:36–44. doi: 10.1016/j.expneurol.2015.07.024 (PMC4654430; doi:10.1016/j.expneurol.2015.07.024)
Supplement: Supplemental Table 3 — Coefficient of variation (CV) and coefficient of error of the mean (CE) for stereological assessment of TH-positive neurons in the substantia nigra. [file mmc3.docx]

Supplemental Table 3

Coefficient of variation (CV) and coefficient of error of the mean (CE) for stereological assessment of TH-positive neurons in the substantia nigra

| Group | CV | CE |
| --- | --- | --- |
| vehicle + saline | 10.28 | 5.2 |
| DFU + saline | 10.60 | 4.8 |
| JZL184 + saline | 9.29 | 5.13 |
| DFU+ JZL184 + saline | 10.08 | 4.8 |
| vehicle + MPTP | 7.96 | 7.11 |
| DFU + MPTP | 11.93 | 6.33 |
| JZL184 + MPTP | 10.59 | 5.64 |
| DFU+ JZL184 + MPTP | 9.51 | 5 |
